# Use of Nonabsorbable Spiral Tacks for Mesh Reinforcement in Thoracoscopic Repair of Congenital Diaphragmatic Hernia

**DOI:** 10.1055/s-0037-1612618

**Published:** 2018-03-22

**Authors:** Anna Poupalou, Celine Vrancken, Erwin Vanderveken, Henri Steyaert

**Affiliations:** 1 Department of Pediatric Surgery/ Pediatrics, Université Libre de Bruxelles (ULB), Hôpital CHU St Pierre, Bruxelles, Belgium; 2 ChirurgiePédiatrique, Université Libre de Bruxelles (ULB), Hôpital HUDERF, Bruxelles, Belgium

**Keywords:** diaphragmatic hernia, thoracoscopy, patch, tack

## Abstract

Thoracoscopic prosthetic repair of congenital diaphragmatic hernia (CDH) is a well-established and safe technique in experienced hands but the patching procedure is technically demanding and time consuming. To address the challenges associated with this process (confined working space and restricted time), the aim of this article is to assess the potential improvements in feasibility, efficacy, and safety of patch fixation by using nonabsorbable helicoidal tacks in neonates and infants for the repair of large CDH by thoracoscopy. The new technique has all the advantages of minimal invasive surgery in very young children combined with the advantages of reduced operating time and increased simplicity, and may be a good option in cases of recurrence.

## Introduction

Primary repair or the technically more demanding patching by thoracoscopy is increasingly used for the treatment of diaphragmatic hernia in neonates and children. This minimal invasive approach not only lessens postoperative pain and hospital stay, but also seems to be associated with a better cosmetic outcome and considerably less psychological, anatomical, and functional consequences of a thoracotomy, especially in young patients. 1 2 3

This procedure requires advanced surgical skills and a high level of training using miniature endoscopic instruments, especially when treating the neonatal population. Limited working space and time may result in poor fixation leading to hernia recurrence which remains a major concern for thoracoscopic surgeons operating on these challenging conditions. 1 2 This complication, namely inappropriate fixation and hernia recurrence, is believed to significantly affect morbidity and mortality. 4

In recent years, the advent of new instrumentation and techniques, such as nonabsorbable tack devices, has had a significant impact on surgical practice, notably in the field of hernia in adults (inguinal/umbilical/incisional). 5 6 The ProTack 5mm fixation device is a single-use tool for fastening prosthetic material, such as hernia mesh, to soft tissue. The tack is helical and made of titanium.

In this article, we report two cases in which most of the challenges posed by thoracoscopic prosthetic repair of congenital diaphragmatic hernia (CDH) were easily solved with the use of the ProTack device. The overall procedure was similar to the thoracoscopic prosthetic repair of CDH described in the literature. 2 3 4 This report highlights the possibility of adding spiral tacks to the thoracoscopic pediatric surgeon' armamentarium as an alternative to suturing when patching is necessary.

## Case Reports

### Case 1

A male newborn was delivered vaginally at 36 weeks of gestation. Routine antenatal ultrasound was normal. The baby was placed on a respirator 20 hours after birth because of respiratory distress. A plain radiograph (RX) demonstrated a large left-sided CDH with air-filled loops of bowel filling the entire left thoracic cavity, leading to a significant right mediastinal shift.

**Fig. 1:**
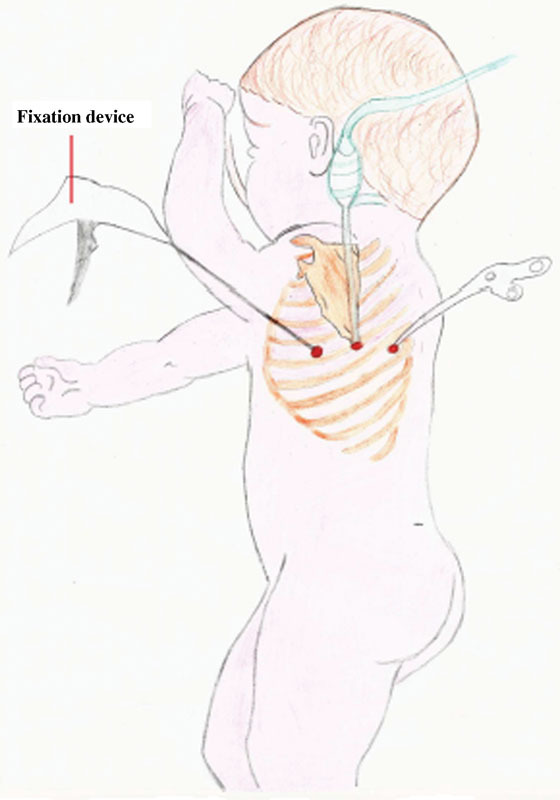
Sites of port placement.

The patient was stabilized on a ventilator and operated on 48 hours later. He was set in the right lateral-decubitus position, and three 5-mm trocars were placed in a triangular shape ( Fig. 1 ). Low-pressure insufflation at 4 mm Hg was applied. The diaphragmatic defect was posterolateral. The entire small bowel as well as the spleen was included in the hernia, and all contents were easily lowered into the peritoneal cavity.

**Fig. 2:**
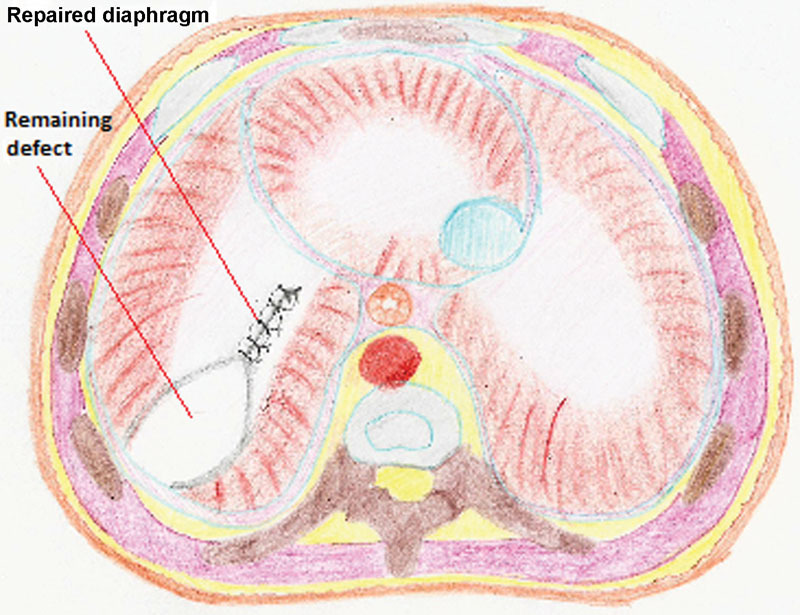
Diaphragmatic defect, partially repaired by thoracoscopic suturing.

**Fig. 3:**
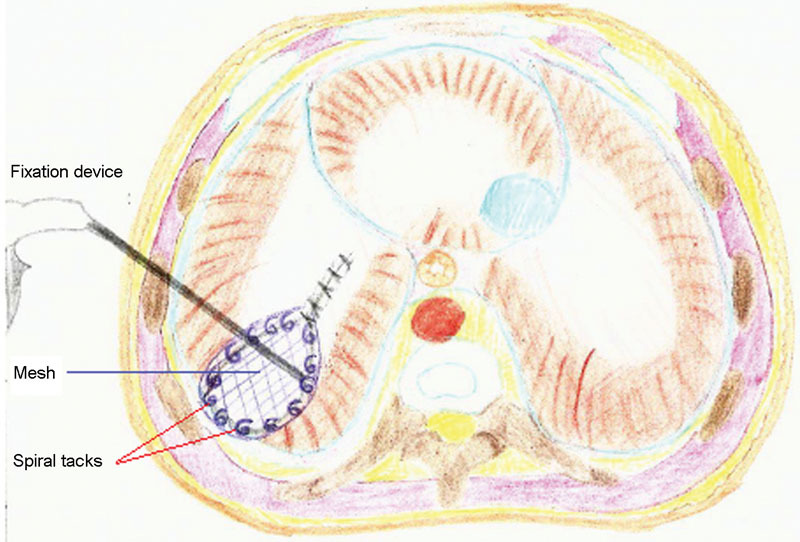
Use of an appropriately sized mesh to close the diaphragmatic defect. The mesh is fixated to the diaphragm using spiral tacks.

**Fig. 4:**
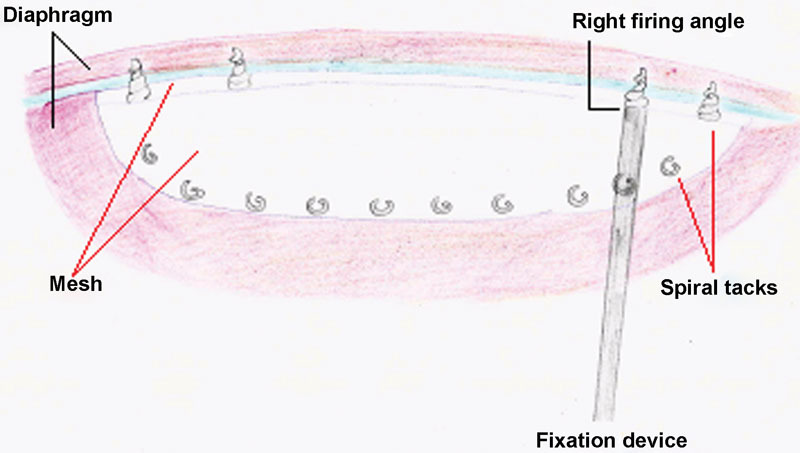
Technique of anchorage of the mesh. It is crucial to secure circumferentially the mesh and deploy tacks perpendicular to the tissue, which can sometimes be challenging.

The posterior edge of the diaphragm was mobilized by incising the overlying peritoneum, and a primary repair was attempted by means of 4–0 braided polyester interrupted sutures knotted intracorporeally ( Fig. 2 ). However, primary approximation of the diaphragm failed because of excessive tension, and the use of a prosthetic patch (Parietex Composite Mesh, Covidien, Ireland [Protack 5 mm, Covidien,Mansfield, USA]) was deemed necessary. Once adjusted in size to match the defect, the mesh was secured to the diaphragm and directly to the ribs (where the diaphragm was completely absent) using seven nonabsorbable spiral tacks (ProTack 5mm, Covidien) ( Figs. 3 and 4 ). No bleeding from the adjacent vessels and no fracture of the ribs were observed during tack application and a right firing angle was obtained. A chest drain was left in the thoracic cavity at the end of the procedure. Operating time was ∼110 minutes.

**Fig. 5:**
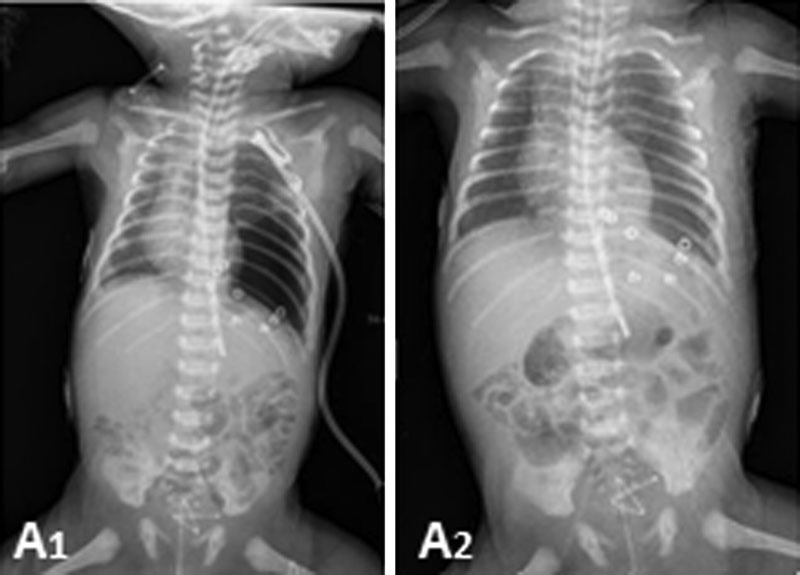
Postoperative chest radiograph: (A1) Immediate postoperative and (A2) Two days postoperatively. The size of the left lung gradually increased, and the mediastinal shift resolved.

The postoperative course was uneventful. The size of the left lung gradually increased, and the mediastinal shift resolved ( Fig. 5 A1 , A2 ). The patient was discharged from the hospital 3 weeks after the operation. He progressed well throughout the follow-up period, presenting a normal chest RX, and the patient remains asymptomatic 4 years and 3 months after surgery.

### Case 2

A 6-month-old boy was admitted to the hospital with acute respiratory distress. He was delivered at 38 weeks of gestation with a birth weight of 3,100 g. Notable medical history included an antenatally diagnosed left-sided CDH associated with extreme pulmonary hypoplasia. He underwent fetal endoscopic tracheal occlusion (Fetendo-PLUG) with limited response. His diaphragmatic defect was repaired on the fifth day of life in another institution and included placement of a patch over a large left posterolateral defect through a classical subcostal left laparotomy. A Nissen fundoplication with gastrostomy was also performed at 3 months of age because of significant gastroesophageal reflux and failure to thrive.

At 6 months of age, acute respiratory distress with cyanosis, tachypnea, lethargy, and opisthotonos were observed at the arrival in the emergency room. After cardiopulmonary resuscitation and artificial ventilation, a chest RX and a three-dimensional computed tomography (CT scan) 2 days later revealed a recurrence of the left CDH with small bowel loops filling the left thoracic cavity.

A three trocar thoracoscopy was scheduled after stabilization and performed as in case 1. With the help of the capnothorax, the hernia contents were gently lowered into the abdomen and pieces of the former patch were extracted. A primary closure seemed impossible due to both a too large posterolateral diaphragmatic defect and very inflamed edges. A nonabsorbable Parietex mesh (Parietex Composite Mesh, Covidien) was carefully positioned and secured in place to cover the diaphragmatic defect. The patch was completely secured to the costal margins and diaphragm with spiral titanium tacks (ProTack 5mm, Covidien). In this case, ∼15 tacks were needed to achieve attachment. No chest tube was left in situ in this case.

The infant recovered well and was discharged from the hospital 1 month after the operation with no recurrence of the hernia on CT scan. After a few months, he was on complete oral feeds. A chest X-ray 1 year post-repair demonstrated a normal thorax, and no evidence of recurrence. Over a 5 ½-year period of follow-up, no subsequent episodes of recurrence occurred, and growth of the child was normal, without complaints.

## Discussion

CDH is a complex, life-threatening malformation which still presents a high morbidity and mortality rate, with survival rate ranging between 50% and 80% in the different studies. 7 In the last decades, prodigious effort has been made to improve the treatment of patients with CDH. 7 8

Laparoscopy was, naturally, the first minimal invasive approach for CDH repair. 9 To date, no studies comparing laparoscopic and thoracoscopic techniques have been performed, but the latter offers advantages in terms of space and easy reduction in the abdominal content, and has been preferred for Bochdalek hernias repair by many authors. 9 10 With thoracoscopy, one can directly visualize the herniated viscera, gently push it into the peritoneal cavity, and then observe and repair the diaphragmatic defect with or without patching. 9

Despite progress in instrumentations and improved surgeon' skills, repair of large CDH defects through thoracoscopic prosthetic repair is still significantly challenging due to the difficulty of intrathoracic suturing. Excessive perioperative hypercapnia and prolonged postoperative low brain oxygenation, along with difficult patch fixation and hernia recurrence, remain the most important factors affecting postoperative morbidity and mortality. 4 9 11 Different types of intra or extracorporeal knots, interrupted or running sutures, rib-anchoring stitches with or without skin incisions, and the Reverdin needle are some of the suturing methods available. Each one of them has been reported to have advantages and disadvantages. 12 13 The ideal tool that provides a quick and safe patch fixation, without suturing difficulties or failures, has yet to be found.

In this report, nonabsorbable spiral tacks were used for mesh fixation during the thoracoscopic repair of large congenital diaphragmatic defects, allowing for easy, quick, and efficient anchoring of the patch to the edges of the defect or ribs. This method has been applied mainly in laparoscopic inguinal, incisional, and ombilical hernia repair in adults. Dapri et al recently described a case of a nontraumatic left lateral diaphragmatic hernia repair by single-incision laparoscopy (SILS) with mesh reinforcement using spiral tacks in a 45-year-old male. 14 A case of thoracoscopic repair of recurrent diaphragmatic hernia using spiral tacks to fix the mesh in a neonate was also reported recently by Riquelme et al. 15 In our cases, no intraoperative complications, bleeding, or abnormal positioning occurred during tack application. In addition, no displacement of the tacks and no hernia recurrence were observed in the immediate postoperative period suggesting that the anchoring of the mesh was reliable. The presence of the helicoidal tacks in the anatomic position of the diaphragmatic defect without any damage to adjacent structures was confirmed through imaging studies during the follow-up period. In the remaining pulmonary parenchyma, no eventration or herniation was seen up to 4 years and 3 months, and 5 ½ years postoperatively for the first and second cases, respectively, indicating that the repair of the diaphragm was definitive and the placement of the tacks appropriate.

In both cases, we placed the spiral tacks with care to ensure their stability and to minimize protrusion. This procedure was challenging because of the small size of the operating field. It was sometimes necessary to change the position of the ProTack device, to achieve better placement of the tacks. As these devices are seldom used in the thorax, we used suggestions and information in the literature from adults laparoscopic procedures using ProTacks. Despite the widespread use of tacker mesh fixation in the repair of hernias worldwide in adults, only a few complications are reported, such as adhesion, small bowel obstruction, and perforation or volvulus. 16 17 Erosion, perforation, displacement, and hemorrhage could be potential complications in our cases. Careful placement of foreign bodies to ensure their stability and to minimize protrusion or contact with important and fragile structures seems to be the key to decrease the risk of displacement or erosion of the hardware, which could lead to serious complications. Newly designed deployment instruments incorporate an articulating shaft or a hinge mechanism allowing for improved access to different parts of the cavity, and delivering perpendicular placement of tacks more easily. 18 Ideally, the size of these devices should be adapted to the pediatric population.

Absorbable tacks offer an alternative to the nonabsorbable metal ones. 5 The risk of recurrence after mesh fixation by absorbable tacks has been found to be similar to that of nonabsorbable devices by some reports, but it was increased in other series. 5 6 With resorbable devices, the foreign material, which is potentially associated with severe complications, does not remain in the body permanently. Only one case reported a complication due to absorbable tackers used in thoracoscopic CDH repair, which was probably due to suboptimal application. 19 In an experimental study, spiral titanium tacks provided better fixation than absorbable tacks at both perpendicular and acute angles and should be strongly considered when perpendicular tack deployment cannot be achieved like in diaphragmatic hernia repair. 20 Glue mesh fixation is also well known in laparoscopic inguinal hernia repair. In a meta-analysis, glue mesh fixation was compared with spiral tacks in 1,001 patients. 21 There were fewer risks for developing chronic groin pain in the “glue” group. Our two patients have not complained of any pain. The use of this material has been already reported in neonatal cases as sealants over esophageal anastomosis and was found to be both safe and effective. 22

## Conclusion

This report of the two cases indicates that mesh fixation using spiral tacks by means of thoracoscopy in the repair of large CDH in very young children is technically feasible even in case of CDH recurrence. The ProTack device offers a simple, quick, and safe method to achieve excellent fixation. Although no major complications were observed in our cases, a larger experience with this device is needed to recommend its use.
